# Perinatal mental health around the world: priorities for research and service development in South America

**DOI:** 10.1192/bji.2020.9

**Published:** 2020-11

**Authors:** Marta B. Rondon

**Affiliations:** Consultant Psychiatrist, National Institute for Maternal and Perinatal Health (INMP), Lima, Peru. Email: mbrondon@gmail.com

**Keywords:** South America, perinatal psychiatry, research priority, services

## Abstract

Research on the topic of poor perinatal mental health in South America is scarce. Nevertheless, studies have shown that it is not uncommon, and that it is linked to women's experience of sexual and intimate partner violence and to inequality, poverty and low educational attainment. High-quality research in large samples with rigorous methodology is a priority, so that data from this region may be compared and analysed in systematic reviews. The links with intimate partner violence need to be explored. Risk and protective factors must be investigated with a strong intercultural perspective. Service integration needs to be implemented. This will require improvements in the availability, accessibility and quality of obstetric and mental health services. There is a need for targeted evidence-based interventions for women and children at risk that incorporate a strong gender and rights perspective.

Perinatal mental health is a modern concept that brings together newly acquired awareness of the relationship between the quality of care and nurturing in the early years of life and health in adulthood. Customarily based on developmental, attachment and systems theory frameworks, this concept encompasses the well-being of parents and infants from conception to 1 or 2 years of age.^1^ It is unfortunate that maternal mortality and extreme morbidity are still serious concerns in South America, despite promising rates of economic growth. In this context, the lack of prioritisation of the psychosocial and mental aspects of perinatal care is obvious, in spite of epidemiological evidence of the high prevalence of maternal perinatal affective disorders.^2^

## Regional prevalence of perinatal mental illness and associated risk factors

As in other low- and middle-income countries (LMICs), perinatal affective disorders are more common in South America than in high-income countries. In 2012, Fisher and her group reviewed the literature on maternal pre- and postnatal mental health from 17 LMICs.^3^ They report a pooled prevalence of 15.9% (95% CI 15.0–16.8%) for antenatal depression and 19.8% (95% CI 19.2–20.6) for postnatal depression. More recently, colleagues and I reviewed the literature and found a pooled prevalence for antenatal depression of 25.3% (95% CI 21.4–29.6) from 51 papers (17 of which came from Latin America).^4^ The pooled prevalence rate for postpartum depression was 19.0% (95% CI 15.5–23.0%) from 53 papers (11 from Latin America). Both reviews describe classic risk factors, such as previous depression history, obstetric complications and other illnesses concomitant with the pregnancy, poverty and low socioeconomic standing at the time of pregnancy, as would be expected. Furthermore, childhood maltreatment, exposure to intimate partner violence and pregnancy intendedness are identified as risk factors for perinatal mental illness.

Published rates of postpartum depression have been correlated with indices of national health (infant mortality rate, lifetime risk of maternal death, total fertility rate and percentage of low birth-weight infants) and with economic (Gini index) and gross domestic product (GDP) indicators.^5^ These include the proportion of women working 40 or more hours/week, provisions for paid and unpaid maternity leave, and sociodemographic variables (percentage of children living in one-parent families and percentage of infants born outside of marriage). Controlling for these variables yielded a global pooled prevalence of 17.7% (95% CI 16.6–18.8%), but there was a huge variation between countries. Rates were increased by income inequality, maternal mortality, infant mortality and long working hours for the mother (>40 h/week), factors that explained 73% of national variation in the prevalence of postpartum depression.

Recent local research shows, for example, that in Popayan, Colombia, 40.20% (95% CI 33–47%) of women received positive scores when screened for postpartum depression using the Edinburgh Postnatal Depression Scale (EPDS) and the strongest risk factor was poor social support (OR = 12.92, 95% CI 3.61–46.17).^6^

Research in Peru has shown that exposure to violence, both current physical intimate partner violence and sexual violence during childhood, are highly correlated with depression and suicidal ideation during pregnancy,^7^ doubling the risk of depressive symptoms during pregnancy (OR = 2.07, 95% CI 1.58–2.71) and compromising sleeping quality, endocrinological health and obstetric outcomes.

## Sociodemographic factors influencing mental health and healthcare

There are, of course, important contextual factors impinging on sexual and reproductive care and mental health that need to be looked at: South America is a very unequal region afflicted by political instability and high indexes of violence that hamper economic and human development despite vast resources.

Because of the disproportionate power of religious groups and marked social and ethnic differences, traditional values and ways are held fast. Even to this day, only Uruguay allows abortion on demand. Only Brazil, Paraguay, Cuba, Nicaragua, Costa Rica, Colombia, El Salvador, Dominican Republic, Mexico and Ecuador have 80% or more of the demand for family planning support satisfied.^8^

From the standpoint of mental illness, stigma and discrimination still characterise attitudes towards psychiatric care, and patients and mental health problems are considered subjective preoccupations of the well-to-do. This results in scant resources for mental health services, which are not easily accessible, unavailable outside of urban centres, fragmentary and of uneven quality. There has been some progress, on the basis of the 1990 Declaration of Caracas^9^ and driven forward by the Comprehensive Mental Health Action Plan 2013–2020, adopted by the World Health Assembly under resolution WHA66/8.^10^ Chile has advanced the most, in a landmark reform with services available from the primary level and stepped-care schemes. They have shown, for instance, that universal screening for postpartum depression in well-child clinics is possible and is well accepted by women; however, this is not linked to evidence of greater access to treatment.^11^

## Priorities in research and service development

It is clear from the preceding sections that there needs to be more high-quality research on the determinants of pre- and postnatal mental disorders in Latin America. One of the encouraging recent developments in the region has been the Pregnancy Outcomes, Maternal and Infant Cohort Study (PrOMIS), involving collaboration between the T.H. Chan School of Public Health (Harvard University, USA), led by Michelle Williams and Bizu Gelaye, and a group of researchers with the Asociacion Civil Proyectos en Salud (PROESA) in Lima, led by Sixto Sanchez, and the National Institute for Maternal and Perinatal Health, the largest maternity hospital in Peru. Large sample size and strong research skills have produced a large amount of data and several publications dealing with the impact of intimate partner violence on sleep quality, risk of depression, post-traumatic stress disorder, suicidal ideation and inflammatory responses during pregnancy associated with obstetric adverse events such as premature birth and low birth weight. The intricate relationship between the experience of violence and perinatal mental health mandates that the development of interventions to try to break the intergenerational transmission of violence be undertaken seriously. Multisectoral intervention needs to be enlisted to diminish the scourge of violence against women and girls, a major threat to mental health.^12^

The integration of perinatal mental health in general sexual and reproductive services is the logical step in service development, with open flow of information and services with mental health specialists. However, both obstetric care and mental healthcare need to improve in the region: high maternal mortality rates indicate that care is suboptimal and untimely; furthermore, women will continue to be lost to follow-up during the puerperium, unless the well-child clinics are transformed into mother–child centres of care. Therefore, optimization of both mental health and obstetric care is a definite priority in the region with careful attention to overcoming barriers posed by service characteristics and women's knowledge, attitudes and beliefs.

Successful research into risk and protective factors with a strong intercultural lens should allow for the identification of at-risk populations to receive evidence-based interventions. Parenting skills, including affect regulation, reduction of child maltreatment and promotion of democratic interactions in the home should be addressed.

Finally, services need to incorporate a strong gender-sensitive and gender-rights perspective, challenging the prevalent ‘Marianism’ (which glorifies motherhood, while at the same time depicting women as selfless and heroic, willing to put up with the most extreme circumstances in the service of men and children)^13^ with recognition of the different needs of women and of the inequalities they continuously face.^14^ Changes in societal attitudes to motherhood should result in more resources for obstetric care and better recognition of the emotional difficulties women may face in the perinatal period.

## Conclusions

Perinatal mental health has not been significantly researched in South America; violence against women and dire socioeconomic circumstances seem to be implicated by the very high rates of perinatal disorders but research is needed to understand these associations better and to intervene in the most effective way. We need better obstetric services for women, and these should integrate mental healthcare, with especial attention to women and infants at higher risk.
